# Efficacy of artemether–lumefantrine, artesunate–amodiaquine, and dihydroartemisinin–piperaquine for treatment of uncomplicated *Plasmodium falciparum* malaria in Angola, 2015

**DOI:** 10.1186/s12936-017-1712-4

**Published:** 2017-02-02

**Authors:** Mateusz M. Plucinski, Pedro Rafael Dimbu, Aleixo Panzo Macaia, Carolina Miguel Ferreira, Claudete Samutondo, Joltim Quivinja, Marília Afonso, Richard Kiniffo, Eliane Mbounga, Julia S. Kelley, Dhruviben S. Patel, Yun He, Eldin Talundzic, Denise O. Garrett, Eric S. Halsey, Venkatachalam Udhayakumar, Pascal Ringwald, Filomeno Fortes

**Affiliations:** 1 Malaria Branch, Centers for Disease Control and Prevention, 1600 Clifton Road, Atlanta, GA 30329 USA; 2 President’s Malaria Initiative, Centers for Disease Control and Prevention, Atlanta, GA USA; 3 National Malaria Control Program, Ministry of Health, Luanda, Angola; 4 Faculty of Medicine, Agostinho Neto University, Luanda, Angola; 5 Field Epidemiology and Laboratory Training Programme, Ministry of Health, Luanda, Angola; 6 World Health Organization, Luanda, Angola; 7 President’s Malaria Initiative, USAID, Luanda, Angola; 8 Atlanta Research and Education Foundation, Atlanta, GA USA; 9 Global Malaria Programme, World Health Organization, Geneva, Switzerland

## Abstract

**Background:**

Recent anti-malarial resistance monitoring in Angola has shown efficacy of artemether–lumefantrine (AL) in certain sites approaching the key 90% lower limit of efficacy recommended for artemisinin-based combination therapy. In addition, a controversial case of malaria unresponsive to artemisinins was reported in a patient infected in Lunda Sul Province in 2013.

**Methods:**

During January–June 2015, investigators monitored the clinical and parasitological response of children with uncomplicated *Plasmodium falciparum* infection treated with AL, artesunate–amodiaquine (ASAQ), or dihydroartemisinin–piperaquine (DP). The study comprised two treatment arms in each of three provinces: Benguela (AL, ASAQ), Zaire (AL, DP), and Lunda Sul (ASAQ, DP). Samples from treatment failures were analysed for molecular markers of resistance for artemisinin (K13) and lumefantrine (*pfmdr1*).

**Results:**

A total of 467 children reached a study endpoint. Fifty-four treatment failures were observed: four early treatment failures, 40 re-infections and ten recrudescences. Excluding re-infections, the 28-day microsatellite-corrected efficacy was 96.3% (95% CI 91–100) for AL in Benguela, 99.9% (95–100) for ASAQ in Benguela, 88.1% (81–95) for AL in Zaire, and 100% for ASAQ in Lunda Sul. For DP, the 42-day corrected efficacy was 98.8% (96–100) in Zaire and 100% in Lunda Sul. All treatment failures were wild type for K13, but all AL treatment failures had *pfmdr1* haplotypes associated with decreased lumefantrine susceptibility.

**Conclusions:**

No evidence was found to corroborate the specific allegation of artemisinin resistance in Lunda Sul. The efficacy below 90% of AL in Zaire matches findings from 2013 from the same site. Further monitoring, particularly including measurement of lumefantrine blood levels, is recommended.

**Electronic supplementary material:**

The online version of this article (doi:10.1186/s12936-017-1712-4) contains supplementary material, which is available to authorized users.

## Background

Ensuring that current first- and second-line anti-malarials remain efficacious is a key component of the international effort to reduce malaria morbidity and mortality. Following World Health Organization (WHO) guidelines [1], Angolan national treatment policy recommends one of three artemisinin-based combination therapy (ACT) for the treatment of uncomplicated malaria: artemether–lumefantrine (AL), artesunate–amodiaquine (ASAQ), or dihydroartemisinin–piperaquine (DP).

In recent years, strong evidence has accumulated that malaria parasites resistant to artemisinins have emerged and are spreading in certain parts of Southeast Asia [2, 3]. Monitoring and tracking these parasites has been facilitated by identification of mutations in the K13 propeller gene that serve as molecular markers for artemisinin resistance [4].

Currently there is no evidence that artemisinin-resistant parasites have appeared in sub-Saharan Africa, where the majority of global malaria morbidity and mortality is concentrated [5, 6]. No parasites with K13 mutations associated with artemisinin resistance have been detected [6], and ACT efficacies have remained generally high [7–10]. However, past experience with the spread of chloroquine and sulfadoxine-pyrimethamine resistance stresses the importance of continued vigilance [11].

Angola, malaria endemic in its entirety, has occupied a prominent place in recent anti-malarial resistance monitoring in Africa [12]. Its large population of Asian immigrants and possible risk of importation of artemisinin-resistant parasites from that region drew attention following the publication of a controversial dispatch describing a case of apparent artemisinin treatment failure in a Vietnamese immigrant who had lived in Lunda Sul Province [13–15]. Moreover, recent routine efficacy monitoring in Angola has shown some evidence of decreased lumefantrine susceptibility at certain sites. Therapeutic efficacy monitoring conducted in 2011–2013 in the capital city Luanda reported an uncorrected efficacy of AL of 91% in all ages, falling to 76% in a small sub-set of participants under 5 years of age [16]. Therapeutic efficacy monitoring in 2013 in children in Zaire and Uíge Provinces showed high corrected efficacy of DP in both provinces, high AL corrected efficacy in Uíge Province, but a corrected efficacy below 90% in Zaire Province [17]. No K13 mutations were found in either the 2011–2013 Luanda study or the 2013 Zaire/Uíge study, but *pfmdr1* genotypes previously associated with decreased lumefantrine susceptibility were reported in both studies.

Here, the results of the 2015 round of biennial therapeutic efficacy monitoring in Angola are presented from three provinces selected based on past efficacy results: Benguela, Zaire and Lunda Sul.

## Methods

### Study design

**Fig. 1:**
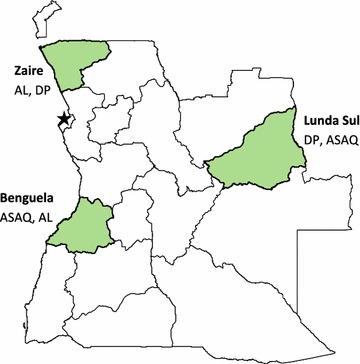
Location of sentinel sites for therapeutic efficacy monitoring in Angola, 2015. *AL* artemether–lumefantrine, *ASAQ* artesunate–amodiaquine, *DP* dihydroartemisinin–piperaquine

Data were collected as part of a six-arm clinical outcomes trial following the standard WHO in vivo therapeutic efficacy protocol [18]. In each of three provinces, the efficacy of two of Angola’s three first-line ACT was measured: AL and DP in the mesoendemic-stable northern province of Zaire, AL and ASAQ in the mesoendemic-stable central coastal province of Benguela, and DP and ASAQ in the hyperendemic eastern province of Lunda Sul (Fig. 1). In each provincial capital, two public health facilities were selected as sentinel sites for patient enrolment. In each province, each anti-malarial regimen was tested consecutively; there was no randomization of participants, and the study was not designed to compare efficacies of the three ACT.

### Study population and enrolment

Children with symptomatic, uncomplicated *Plasmodium falciparum* infection presenting to the sentinel health facilities and meeting standard inclusion criteria [18] were invited to participate in the trial. To account for differences in transmission intensity, the target age range was 6 months to 5 years (59 months) in Zaire and Lunda Sul, and 6 months to 12 years (143 months) in Benguela. Similarly, the acceptable initial parasite density was 2000–200,000 parasites/µL in Zaire and Lunda Sul and 1000–100,000 parasites/µL in Benguela. In addition to parasite density, haemoglobin was measured during enrolment using HemoCue® 201 and 301 devices (AB Leo Diagnostics, Helsinborg, Sweden), with children having haemoglobin >5 g/dL eligible for enrolment.

The target sample enrolment was 100 children per arm, providing enough power to estimate efficacy with 95% confidence limits of ±5% assuming an expected efficacy of 95% and a maximum loss to follow-up and exclusion rate of 27%.

### Study procedures

Study participants were administered one of three anti-malarials: AL (Coartem^®^; Novartis, Basel, Switzerland), ASAQ (Winthrop; Sanofi Aventis, Paris, France), and DP (Duo-cotecxin^®^; Holley-Cotec, Beijing, China). Study participants in the AL arm in Benguela received the regular formulation of Coartem, whereas those in the AL arm in Zaire received the dispersible formulation. AL was procured by The President’s Malaria Initiative and provided by the Angolan National Malaria Control Programme, and the ASAQ and DP were procured and provided by WHO. All medications passed lot-quality testing, performed in laboratories in the USA (AL), the Netherlands (DP) and South Africa (ASAQ), prior to the study. The participants were treated according to manufacturers’ weight-based dosing recommendations. All three doses of ASAQ and DP were directly observed in the study health facilities. For AL, the morning doses were directly observed at the study health facilities, while the evening doses were given by participants’ guardians. To ensure appropriate administration of evening doses, guardians were counselled at the health facility and reminded in the evening by telephone. Guardians were also instructed to bring back empty blister packs the following day. Study participants not correctly completing the medication were withdrawn from the study.

Study participants were followed up daily for the first three days after initiation of treatment, and then weekly thereafter for a total of 28 days (AL and ASAQ arms) or 42 days (DP arms). At each follow-up visit, clinical response to treatment was monitored through a standardized history and physical examination and parasitological response was assessed through preparation and examination of thick blood films (with the exception of day 1). Guardians were asked about any adverse events experienced by the study participants. Blood slides were prepared and read by two dedicated study laboratory technicians following standard procedures [18], and a sub-set of slides was read by expert microscopists from the Angolan National Malaria Control Programme during supervisory visits and following study end. Dried blood spots were collected at enrolment and at all weekly follow-up visits. Additional blood spots were also collected on day 3 in Zaire and Benguela. Follow-up was suspended for all study participants developing any danger signs and/or meeting criteria for treatment failure. Treatment failures were treated with intravenous quinine. An electronic database containing clinical and parasitological data was updated daily using double data entry.

### Molecular analysis

Dried blood spots were transported to CDC laboratories in Atlanta. DNA was extracted using a QIAamp Blood DNA kit (Qiagen, Hilden, Germany) from day 0 and day-of-failure samples for all treatment failures and day-0 samples from 50 randomly selected participants with adequate clinical and parasitological response (ACPR).

For all treatment failure samples and the randomly selected treatment successes, the lengths of six microsatellites [19] were measured: TA1, Polyα, PfPK2, C2M34, C3M69, and 2490. A previously described protocol for cycling [20] was modified for this study. Primer pairs with annealing temperature in the 50–60 °C range were designed and cycling conditions were optimized according to melting temperature for each primer pair. The sizes of the amplified products were assayed by capillary electrophoresis on an Applied Biosystems 3130xl sequencer (Applied Biosystems, Foster City, CA, USA). Microsatellite fragment analysis was performed using the Geneious Pro R8 microsatellite plug-in (Biomatters, San Francisco, CA, USA).

Regions in the *P. falciparum* K13, *P. falciparum* multidrug-resistance gene 1 *(pfmdr1)*, and *P. falciparum* cytochrome b *(pfcytb)* genes were amplified and sequenced for day 0 and day of failure for all treatment failures. The *P. falciparum* K13 propeller domain was amplified using a *P. falciparum*-specific protocol described previously [21]. Two regions of *pfmdr1,* covering codons 86–184 and 1034–1246, were amplified using a previously described protocol [22]. The *pfcytb* gene was amplified using the following primers: forward primer, 5′ CTATTAATTTAGTTAAAGCACAC 3′ and reverse primer, 5′ ACAGAATAATCTCTAGCACCA 3′. 1 µL of genomic DNA was amplified using 0.5 µM of each primer, 0.2 mM dNTP, 2 mM MgCl_2_ and 1 U High Fidelity Taq (New England Biolabs, Ipswich, MA, USA). For the primary reaction, the following cycling parameters were used: 2 min at 98 °C, 35 cycles of 98 °C for 10 s, 46 °C for 30 s, 68 °C for 2:30 min, and final extension for 10 min at 68 °C. Amplified PCR products were visualized on a 2% agarose gel after electrophoresis. Sequencing of PCR products was performed using an ABI 3730 sequencer (Applied Biosystems, Foster City, CA, USA).

Sanger sequences were analysed using Geneious R8 software (Biomatters, San Francisco, CA, USA) to identify specific single-nucleotide polymorphism combinations. Single-nucleotide polymorphisms were only called if both the forward and reverse strands had the mutation.

### Statistical analysis

Study participants not meeting any exclusion criteria were classified as one of three possible study endpoints. Study participants showing signs of severe disease while parasitaemic in the first 3 days following treatment, having a parasite density on day 2 that exceeded day 0 parasite density, clearing less than 75% of day 0 parasites on day 3, or still febrile (≥37.5 °C axillary) and parasitaemic on day 3 were classified as early treatment failures (ETFs). Study participants that were febrile and parasitaemic after day 3 or parasitaemic regardless of body temperature after day 6 were classified as late treatment failures (LTFs). Participants not meeting these criteria with a negative slide on the last day of follow-up were classified as having an ACPR.

Kaplan–Meier estimates of the survival curve for each arm were calculated and plotted. Uncorrected efficacy was calculated as the proportion ACPR (per-protocol method), as well as the Kaplan–Meier estimate of the survival function at day 28 and/or 42. Microsatellite data for LTFs were analysed using a previously published statistical algorithm which assigns each LTF a posterior probability of recrudescence [23]. LTFs with a posterior probability of recrudescence >0.5 were classified as likely recrudescences; all others were classified as re-infections. The point estimates and 95% confidence intervals of the PCR-corrected efficacies were calculated by sampling from the posterior distribution of classifications to account for the uncertainty of the correction [23]. Statistical analysis was performed using R version 3.0.1 (R Foundation for Statistical Computing, Vienna, Austria).

### Ethical considerations

Parents or guardians of all study participants provided written informed consent prior to enrolment and were reimbursed for transport costs for clinic visits. The activity was classified as non-research by human subjects research boards at CDC and the Angolan Ministry of Health and was approved by the WHO Ethical Research Committee.

## Results

A total of 1804 children were screened, of which 586 (32%) were enrolled (Table 1). Five of the six arms reached the 100 participant target; enrolment in the ASAQ arm in Lunda Sul was stopped at 78 children due to a diminishing rate of patient eligibility following the start of the dry season. Loss to follow-up rates ranged from 7% in the AL arm in Zaire to 20% in the ASAQ arm in Benguela, which experienced severe floods during the study period (Table 2). A total of 46 study participants were excluded including: 21 participants who failed to complete treatment, seven who developed a concomitant disease in the absence of parasitaemia, 14 who did not meet inclusion criteria but were erroneously enrolled, two with necessary information not recorded on the form, one with *Plasmodium vivax* infection during follow-up, and one participant in the DP arm in Lunda Sul that developed severe malaria and died fewer than 24 h after enrolment. The minimal sample size of 73 study participants reaching a study endpoint was achieved in all arms except for the ASAQ arm in Lunda Sul, where only 56 participants finished 28-day follow-up. Participant characteristics at baseline reflected the different inclusion criteria in each site (Table 1).

**Table 1:** Number of participants screened, enrolled, and finishing follow-up and characteristics at baseline as part of therapeutic efficacy monitoring in Angola, 2015

	Benguela		Zaire		Lunda Sul	
	ASAQ^a^	AL^a^	AL^a^	DP^b^	DP^b^	ASAQ^a^
Screened, *n*	445	443	150	223	318	225
Enrolled, *n*	101	101	104	102	100	78
Lost to follow up, *n* (%)	20 (20)	11 (11)	7 (7)	12 (12)	14 (14)	9 (12)
Excluded, *n* (%)	8 (8)	5 (5)	4 (4)	6 (6)	10 (10)	13 (17)
Reached study endpoint, *n* (%)	73 (72)	85 (84)	93 (89)	84 (82)	76 (76)	56 (72)
Participant characteristics at baseline						
Median age, years (range)	5.0 (0.7–12)	6.4 (1–12)	3.0 (0.6–5)	2.8 (0.6–5)	2.8 (0.5–5)	3.2 (0.5–5)
Median weight, kg (range)	16 (6–37)	18 (8–42)	13 (7–19)	13 (6–19)	12 (6–21)	12 (7–18)
Percent female	40%	40%	51%	53%	42%	58%
Median day 0 parasitemia, parasites/µL (range)	29,487 (1051–99,507)	20,151 (1003–92,733)	47,766 (2034–195,529)	48,049 (2003–187,777)	7266 (2000–180,903)	16,071 (2000–161,333)
Median day 0 hemoglobin, g/dL (range)	10.3 (5.2–13.3)	10.2 (5.4–13.5)	10.2 (5.7–13.6)	10.0 (6–13.3)	9.4 (5.1–13.5)	9.2 (5.5–13.6)

*ASAQ* artesunate–amodiaquine,* AL* artemether–lumefantrine, *DP* dihydroartemisinin–piperaquine

^a^28-day follow-up

^b^42-day follow-up

**Table 2:** Treatment outcomes for participants finishing follow-up as part of therapeutic efficacy monitoring in Angola, 2015

	n (%)					
	Benguela		Zaire		Lunda Sul	
	ASAQ^a^	AL^a^	AL^a^	DP^b^	DP^b^	ASAQ^a^
	*n* = *73*	*n* = *85*	*n* = *93*	*n* = *84*	*n* = *76*	*n* = *56*
Treatment failure	7 (10)	9 (11)	23 (25)	15 (18)	0	0
Early treatment failure	0	1 (1)	3 (3)	0	0	0
Late treatment failure	7 (10)	8 (9)	20 (22)	15 (18)	0	0
Recrudescence	0	1 (1)	8 (9)	1 (1)	0	0
Day 7	0	0	0	0	0	0
Day 14	0	0	2 (2)	0	0	0
Day 21	0	0	6 (6)	0	0	0
Day 28	0	1 (1)	0	1 (1)	0	0
Day 35	–	–	–	0	0	–
Day 42	–	–	–	0	0	–
Reinfection	7 (10)	7 (8)	12 (13)	14 (17)	0	0
Day 7	0	0	0	0	0	0
Day 14	0	2 (2)	1 (1)	0	0	0
Day 21	1 (1)	1 (1)	6 (6)	0	0	0
Day 28	6 (8)	1 (1)	5 (5)	3 (4)	0	0
Day 35	–	–	–	5 (6)	0	–
Day 42	–	–	–	6 (7)	0	–
Adequate clinical and parasitological response	66 (90)	76 (89)	70 (75)	69 (82)	76 (100)	56 (100)

*ASAQ* artesunate–amodiaquine, *AL* artemether–lumefantrine, *DP* dihydroartemisinin–piperaquine

^a^28-day follow-up

^b^42-day follow-up

All medications were generally well tolerated. Vomiting following administration of the drug occurred in 3.9% (7/179) of participants taking ASAQ, 7.3% (15/205) of participants taking AL, and 8.4% (17/202) of participants taking DP. One participant in the DP arm in Zaire experienced seizures on day 12 of follow-up, was negative for malaria by microscopy, and was excluded. No other adverse events were reported.

Clearance of parasites in all six arms was rapid, with clearance rates in each arm ranging from 94 to 100% on day 2, increasing to 99 to 100% on day 3 (Table 3). Four ETFs were reported. Three of the ETFs occurred in the AL arm in Zaire; in all three cases, the clinical status of the child worsened on day 1, and the child’s haemoglobin was measured to have fallen below 5 g/dL. Haemoglobin at enrolment in these study participants ranged from 6.2 to 7.8 g/dL.

**Table 3:** Proportion of slides negative for asexual malaria parasites on day 2 and 3 following anti-malarial treatment, therapeutic efficacy monitoring in Angola, 2015

	Proportion slides negative (95% confidence intervals)					
	Benguela		Zaire		Lunda Sul	
	ASAQ	AL	AL	DP	DP	ASAQ
Day 2	99 (93–100)	98 (92–100)	95 (87–98)	94 (87–97)	100 (93–100)	100 (90–100)
Day 3	100 (95–100)	100 (95–100)	99 (93–100)	99 (94–100)	100 (92–100)	100 (89–100)

*ASAQ* artesunate–amodiaquine,* AL* artemether–lumefantrine, *DP* dihydroartemisinin–piperaquine

The Kaplan–Meier survival curves were markedly different for the six arms, with the Benguela ASAQ and both Lunda Sul arms showing maintained high efficacy over time, in contrast to the Benguela AL and both Zaire arms showing declining efficacy during follow-up (see Additional file 1: Figure S1). Overall, the uncorrected 28-day efficacy using the Kaplan–Meier estimate of the survival curve was 90.3% (95% CI 84–97%) for the ASAQ arm in Benguela, 89.6% (83–96%) for the AL arm in Benguela, 76.0% (68–85%) for the AL arm in Zaire, 95.5% (91–100%) for the DP arm in Zaire, 100% (94–100%) for the DP arm in Lunda Sul, and 100% for the ASAQ arm in Lunda Sul (Table 4). The uncorrected 42-day efficacy of DP was 82.5% (75–91%) in Zaire and 100% (94–100%) in Lunda Sul. The efficacies using the proportion ACPR indicator were not significantly different from the Kaplan–Meier estimate.

**Table 4:** Efficacy of first-line anti-malarials in three therapeutic efficacy monitoring sites in Angola, 2015

	Efficacy (95% confidence intervals)					
	Benguela		Zaire		Lunda Sul	
	ASAQ^a^	AL^a^	AL^a^	DP^b^	DP^b^	ASAQ^a^
Uncorrected						
Per-protocol Day 28	90.4 (81–96)	89.4 (80–95)	75.3 (65–83)	95.6 (88–99)	100 (94–100)	100 (92–100)
Per-protocol Day 42	–	–	–	81.9 (72–89)	100 (94–100)	–
Kaplan–Meier estimate Day 28	90.3 (84–97)	89.6 (83–96)	76.0 (68–85)	95.5 (91–100)	100	100
Kaplan–Meier estimate Day 42	–	–	–	82.5 (75–91)	100	–
PCR-corrected						
Per-protocol Day 28	99.9 (95–100)	96.1 (89–99)	86.5 (77–92)	98.8 (94–99)	100 (96–100)	100 (94–100)
Per-protocol Day 42	–	–	–	98.5 (92–99)	100 (96–100)	–
Kaplan–Meier estimate Day 28	99.9 (95–100)	96.3 (91–100)	88.1 (81–95)	98.8 (96–100)	100	100
Kaplan–Meier estimate Day 42	–	–	–	98.8 (96–100)	100	–

Per-protocol efficacy defined as proportion adequate clinical and parasitological response (ACPR), Kaplan–Meier estimate calculated from estimate of survival function

*ASAQ* artesunate–amodiaquine, *AL* artemether–lumefantrine, *DP* dihydroartemisinin–piperaquine

^a^28-day follow up

^b^42-day follow up

Of the 50 LTFs, 40 were reclassified as likely re-infections and ten as likely recrudescences. Due to high allelic diversity, there was little uncertainty around the classification, with 44 of 50 (88%) LTFs having a probability of recrudescence either >0.9 or <0.1, signifying high confidence (see Additional file 2: Table S1). The majority of recrudescences were observed in the AL arm in Zaire. The corrected 28-day Kaplan–Meier efficacy estimates were 99.9% (95–100%) for the ASAQ arm in Benguela, 96.3% (91–100%) for the AL arm in Benguela, 88.1% (81–95%) for the AL arm in Zaire, 98.8% (96–100%) for the DP arm in Zaire, 100% for the DP arm in Lunda Sul, and 100% for the ASAQ arm in Lunda Sul (Table 4). For DP, the corrected 42-day efficacies were 98.8% (96–100%) in Zaire and 100% in Lunda Sul.

All treatment failures were wild type for K13 and *cytb* (Table 5). The *pfmdr1* gene, however, showed more diversity, in particular in the polymorphic 86, 184 and 1246 codons. Of the five observed *pfmdr1* haplotypes, NYD (86N, 184Y, 1246D) and NFD (86N, 184F, 1246D) were the most common haplotypes, found in 61 (59%) and 33 (32%) of tested samples, respectively. In the AL arms, the recrudescent and incident re-infections were all either NYD (61%, 17/28) or NFD (39%, 11/28).

**Table 5:** Molecular markers of resistance genotypes for treatment failures observed during therapeutic efficacy monitoring in Angola, 2015

Marker	n (%)															
	Benguela							Zaire								
	ASAQ		AL					AL					DP			
	*n* = *7*	*n* = *7*	*n* = *1*	*n* = *7*	*n* = *7*	*n* = *1*	*n* = *1*	*n* = *3*	*n* = *12*	*n* = *12*	*n* = *8*	*n* = *8*	*n* = *14*	*n* = *14*	*n* = *1*	*n* = *1*
	Reinf day 0	Reinf day failure	ETF	Reinf day 0	Reinf day failure	Recr day 0	Recr day failure	ETF	Reinf day 0	Reinf day failure	Recr day 0	Recr day failure	Reinf day 0	Reinf day failure	Recr day 0	Recr day failure
K13																
Wildtype	7 (100)	7 (100)	1 (100)	7 (100)	7 (100)	1 (100)	1 (100)	3 (100)	12 (100)	12 (100)	8 (100)	8 (100)	14 (100)	14 (100)	1 (100)	1 (100)
*cytB*																
Wildtype	7 (100)	7 (100)	1 (100)	7 (100)	7 (100)	1 (100)	1 (100)	3 (100)	12 (100)	12 (100)	8 (100)	8 (100)	14 (100)	14 (100)	1 (100)	1 (100)
*pfmdr1* ^a^																
NYD	5 (71)	5 (71)	1 (100)	5 (71)	5 (71)	–	–	1 (33)	7 (58)	7 (58)	5 (62)	5 (62)	7 (50)	8 (57)	–	–
NFD	1 (14)	1 (14)	–	2 (29)	2 (29)	1 (100)	1 (100)	–	3 (25)	5 (42)	3 (38)	3 (38)	5 (36)	4 (29)	1 (100)	1 (100)
YFD	–	–	–	–	–	–	–	–	–	–	–	–	1 (7)	1 (7)	–	–
YYD	1 (14)	–	–	–	–	–	–	2 (67)	2 (17)	–	–	–	1 (7)	1 (7)	–	–
YYY	–	1 (14)	–	–	–	–	–	–	–	–	–	–	–	–	–	–

In addition to the asynonymous mutations listed in the table, the following synonomous mutations were also observed: *pfmdr1* G102G (2 instances), *pfmdr1* T1069T (15), *pfmdr1* S1137S (1), *cytB* A295A (1), K13 P553P (1), K13 T535T (2)

*ETF* early treatment failure, *Reinf* reinfection, *Recr* recrudescence, *ASAQ* artesunate–amodiaquine, *AL* artemether–lumefantrine, *DP* dihydroartemisinin–piperaquine

^a^ *pfmdr1* haplotype classified according to animo acids at positions 86, 184, and 1246

## Discussion

These therapeutic efficacy monitoring results from the three provinces demonstrate that the artemisinin components of the three ACT in use in Angola are still highly effective against falciparum malaria, while there is some variation between the efficacies of the partner drugs by site. Although the three sentinel sites are dispersed throughout the country (Fig. 1), the efficacy results are not necessarily generalizable to the entire country.

No K13 mutations were detected in any of the treatment failures. While there is no guarantee that artemisinin resistance in sub-Saharan Africa will necessarily be mediated by K13 mutations [24], the absence of K13 mutations, together with high clearance rates in all arms, is strong evidence of a circulating parasite population highly susceptible to artemisinin derivatives in the sentinel sites. Most of the ETFs occurred in children becoming severely anaemic (haemoglobin ≤5 g/dL) in the first two days following treatment. The occurrence of this in study participants with already low haemoglobin at enrolment might argue for changing the haemoglobin criteria at enrolment. Currently, the standard WHO in vivo therapeutic efficacy monitoring protocol recommends a cut-off for haemoglobin of 5 g/dL, but the results here argue for adoption of more stringent criteria for future therapeutic efficacy monitoring, for example haemoglobin >8 g/dL at enrolment.

Although the ASAQ arm in Lunda Sul did not meet its target sample size, the high efficacy of both DP and ASAQ in Lunda Sul, the absence of K13 mutants, and high clearance rates provide no evidence that a focus of artemisinin resistance has been established in Lunda Sul.

In general, all three of the partner drugs to the artemisinin derivatives were efficacious in sufficiently suppressing the initial malaria infection. No recrudescences were observed in either the Benguela or the Lunda Sul ASAQ arm. A total of 15 LTFs were observed in the DP arms, with all of these occurring in the Zaire arm. The 42-day uncorrected efficacy of DP in Zaire was estimated to be 82.5%, which is lower than expected, as 42-day uncorrected efficacies of DP are usually reported to be closer to 90% [25], a result possibly explained by an unusually high transmission intensity in the Zaire site. Eleven (73%) of the LTFs in the Zaire DP arm were past 28 days of follow-up. All of these were re-infections, as the only recrudescence occurred at day 28. As a result, the corrected 28- and 42-day efficacies for DP were virtually identical and approaching 100% in both Zaire and Lunda Sul.

The AL arms recorded a total of 38 LTFs, despite only a 28-day follow-up duration, resulting in 28-day uncorrected efficacies below 90% in both Benguela and Zaire. This was likely partly due to lumefantrine having a significantly shorter half-life than piperaquine [26]. The longer prophylactic effect of DP, confirmed here, should perhaps be factored in policy decisions in Angola, as recent modelling work has postulated a reduction in malaria incidence in high transmission areas in Africa if DP were to be used in place of AL [27].

Amongst the 38 LTFs observed in the AL arms, nine were classified as recrudescent infections. While only one of the LTFs in Benguela was a recrudescence of the original infection, there were eight recrudescences noted in the AL arm in Zaire. Combined with three ETFs, this resulted in a corrected efficacy, using the Kaplan–Meier estimator, of 88.1% in Zaire, close to the 89.6% estimate reported in 2013 [17]. It is also close to the efficacy estimate of 91.3% in 2011–2013 in Luanda. However, comparison of these efficacies is complicated by different upper age limits of the study participants: up to five years in Zaire in 2015, up to nine years in Zaire in 2013, and all ages in Luanda in 2011–2013. Nevertheless, the results are a continuation of the trend of AL efficacy in Zaire below the 90% lower limit of efficacy recommended for ACT, and are cause for concern.

The preponderance of *pfmdr1* NYD and NFD haplotypes in AL recrudescent and incident infections during follow-up closely matches data from the 2013 therapeutic efficacy monitoring in Zaire and Uíge Provinces, where 91% of AL treatment failures had NYD or NFD haplotypes on day of failure. This is consistent with evidence that NYD and NFD haplotypes are associated with decreased susceptibility to lumefantrine and are under selection following AL treatment [22, 28, 29]. There was no evidence of selection of K13 mutants over the course of treatment, as all day-0 and day-of-failure isolates had wild type K13 genotypes.

The fact that lumefantrine drug levels were not measured and that the evening AL doses were not directly observed precludes the exclusion of underdosing or malabsorption as explanations for the decreased efficacy of AL in Zaire Province. Future therapeutic efficacy monitoring should incorporate direct observation of all doses of AL, including strict adherence to food intake during administration of AL, and measurement of day 7 lumefantrine levels to definitively rule out the alternative hypothesis of underdosing or malabsorption.

The sequential, non-randomized enrolment of study participants meant that participants in the different arms were exposed to different risk of re-infection as the follow-up period in the second arm in each province took place later in the peak transmission season. As a result, direct comparison of drug efficacies is difficult across study arms.

## Conclusion

Overall, the 2015 round of efficacy monitoring in Angola confirms the continued sensitivity of *P. falciparum* parasites to artemisinin derivatives, points to high efficacy of amodiaquine and piperaquine as partner drugs, and reaffirms a low efficacy of lumefantrine in Zaire Province in northern Angola. Further routine efficacy monitoring, including direct observation of all doses and blood drug level measurement, is recommended.

## Additional files

**Additional file 1.** Kaplan–Meier estimates of the proportion of participants with adequate clinical and parasitological response (ACPR) over the course of follow up during therapeutic efficacy monitoring in Angola, 2015. AL: Artemether-lumefantrine, ASAQ: Artesunate-amodiaquine, DP: Dihydroartemisinin-piperaquine.

**Additional file 2.** Lengths of neutral microsatellite markers for late treatment failures and randomly chosen treatment successes, Angola therapeutic efficacy monitoring, 2015.
