# Supplementary material for: Efficacy of artemether–lumefantrine, artesunate–amodiaquine, and dihydroartemisinin–piperaquine for treatment of uncomplicated Plasmodium falciparum malaria in Angola, 2015
Source: Malar J. 2017 Feb 2;16:62. doi: 10.1186/s12936-017-1712-4 (PMC5290657; doi:10.1186/s12936-017-1712-4)
Supplement: Supplementary file 1 — Additional file 1. Kaplan–Meier estimates of the proportion of participants with adequate clinical and parasitological response (ACPR) over the course of follow up during therapeutic efficacy monitoring in Angola, 2015. AL: Artemether-lumefantrine, ASAQ: Artesunate-amodiaquine, DP: Dihydroartemisinin-piperaquine. [file 12936_2017_1712_MOESM1_ESM.pdf]

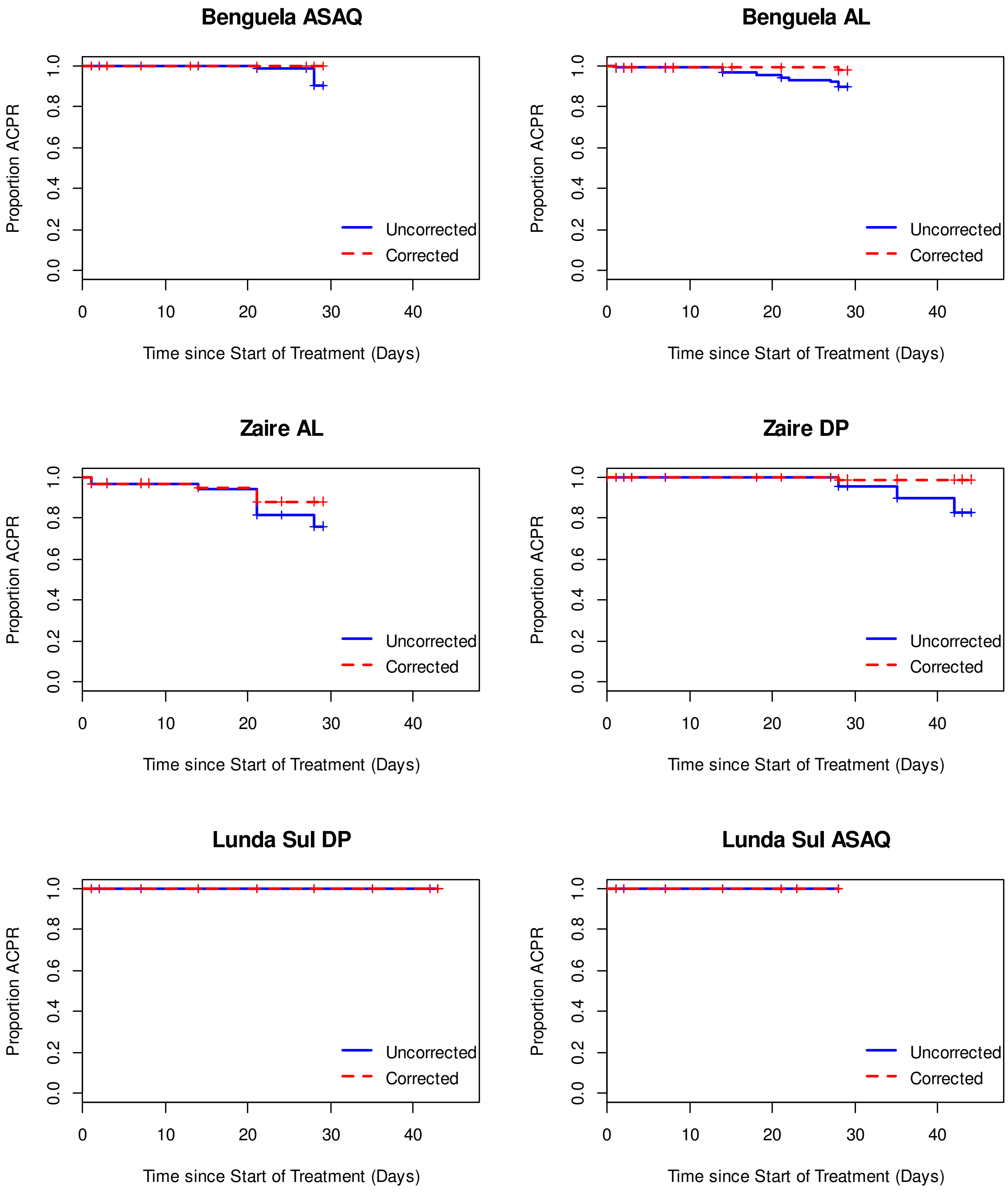

Supplementary Figure S1. Kaplan-Meier estimates of the proportion of participants with adequate clinical and parasitological response (ACPR) over the course of follow up during therapeutic efficacy monitoring in Angola, 2015. AL: Artemether-lumefantrine, ASAQ: Artesunate-amodiaquine, DP: Dihydroartemisinin-piperaquine
