# Supplementary material for: Efficacy of artemether–lumefantrine, artesunate–amodiaquine, and dihydroartemisinin–piperaquine for treatment of uncomplicated Plasmodium falciparum malaria in Angola, 2015
Source: Malar J. 2017 Feb 2;16:62. doi: 10.1186/s12936-017-1712-4 (PMC5290657; doi:10.1186/s12936-017-1712-4)
Supplement: Supplementary file 2 — Additional file 2. Lengths of neutral microsatellite markers for late treatment failures and randomly chosen treatment successes, Angola therapeutic efficacy monitoring, 2015. [file 12936_2017_1712_MOESM2_ESM.pdf]

Supplemental Table 1. Lengths of neutral microsatellite markers for late treatment failures and randomly chosen treatment successes, Angola therapeutic efficacy monitoring, 2015

		Chromosome	2		3			6			4			12					10		Recrudescence
		Tandem Repeats	AT		TA			ATT			ATT			TAA					TAA		Reinfection
		Marker Name	313		383			TA1			POLYA			PFPK2					2490		
Province	Drug	Sample ID	313_1	313_2	383_1	383_2	383_3	TA1_1	TA1_2	TA1_3	POLYA_1	POLYA_2	POLYA_3	PFPK2_1	PFPK2_2	PFPK2_3	PFPK2_4	PFPK2_5	2490_1	2490_2	Probability of Recrudescence
Late Treatment Failures																					
Benguela	AL	BL163_D0	238.4		124.5			175.6			144.7	150.9		172.1					83.6		0.17
Benguela	AL	BL163_D18	241.477		134.775			172.546			154.212			171.557					80.061		
Benguela	AL	BL186_D0	226.3		141.3			160.5			145.6			163.1					83.7		0.99
Benguela	AL	BL186_D28	227.162		139.853			160.309			144.1			0					83.406		
Benguela	AL	BL215_D0	238.6		155.7			169.5			158.2			187					83.7		0.00
Benguela	AL	BL215_D28	233.774	241.767	124.842			181.72			151.2			159.569					73.677	83.389	
Benguela	AL	BL225_D0	234.6		144.1			169.3			166.7			183.6					83.6		0.44
Benguela	AL	BL225_D21	241.377		124.445	147.239		153.582	160.211		154.242	167.826		162.641	184.164	189.861	192.943		83.291	86.562	
Benguela	AL	BL230_D0	240.6		152.5			178.6			155			181.6					83.6		0.00
Benguela	AL	BL230_D28	251.839		124.414	145.167		163.298	166.368		155.012			162.967	171.812				83.307		
Benguela	AL	BL236_D0	217.9		124.5			165.3	177.7		151.8	158.1		172.7					73.8	83.6	0.27
Benguela	AL	BL236_D14	220.706		124.417	145.029		160.224			158.267	186.659	192.92	168.668	172.019				80.086	83.325	
Benguela	AL	BL238_D0	224.1		137.7			172.4			167.6			168.5					83.6		0.05
Benguela	AL	BL238_D14	218.696	244.464	124.435	134.765		160.013	178.436		154.247	158.373	163.928	156.774	171.838				82.391		
Benguela	AL	BL239_D0	224.1	236.2	140.172			163.002			154.583	180.311		162.824					79.9		0.18
Benguela	AL	BL239_D21	241.369		147.213			166.388	169.46		164.632	177.325		162.44	181.099				83.003		
Benguela	ASAQ	BQ602_D0	218.1		153.7			160.2	166.3		155	167.6		159.8	162.6				83.4	86.7	0.00
Benguela	ASAQ	BQ602_D21	226.919		125.399			180.1			160.617			159.521					83.293		
Benguela	ASAQ	BQ603_D0	222.4		123.2			193.9			157.2			144.2					80.2		0.00
Benguela	ASAQ	BQ603_D28	239.617		136.898			177.1			152.07	170.86		159.539					83.309		
Benguela	ASAQ	BQ619_D0	232.3		152.8			169.3			141.3			172.4					83.6		0.01
Benguela	ASAQ	BQ619_D28	221.639	242.382	123.181			169.473	175.611		158.357			162.291	171.644				82.369		
Benguela	ASAQ	BQ637_D0	213.8		123.7			174.5	202.1		141.4	161.4		196.1	199.4				83.6		0.00
Benguela	ASAQ	BQ637_D28	241.409		124.647			166.164			141.665			162.784					83.339		
Benguela	ASAQ	BQ638_D0	224.1		149.2			178.5			154.1			175.1					84		0.01
Benguela	ASAQ	BQ638_D28	233.3		124.45			165.3			142			0					80	83.1	
Benguela	ASAQ	BQ670_D0	220.1		144.7			172.4	181.7		150.9			162.8					73.8	83.6	0.08
Benguela	ASAQ	BQ670_D28	224.976	235.39	136.909			169.564			170.888			171.888					73.521	83.27	
Benguela	ASAQ	BQ680_D0	226.5		142.8			163.3			155			172.3					83.6		0.00
Benguela	ASAQ	BQ680_D28	263.916		124.595			181.728			158.318			171.876					83.23		
Zaire	DP	ZD008_D0	256.8		136.8			184.6			154.1			172.1	174.7				83.5		0.00
Zaire	DP	ZD008_D42	230.521		144.71			184.807			127.7			159.959					83.339		
Zaire	DP	ZD018_D0	238.4		141			171.3			151.8			172.2					83.6		0.00
Zaire	DP	ZD018_D35	257.489		136.919			184.839			179.249			162.7					86.632		
Zaire	DP	ZD020_D0	230.2	244.3	152.6			166			148.7	185.5		165.2					80.3		0.01
Zaire	DP	ZD020_D42	256.529		145.156			163.35			145.589	161.329		159.28	187.165				80.027	83.26	
Zaire	DP	ZD025_D0	246.3		125.3	138.8	143.1	168.3	174.3	195.8	145.5	167.6		175.2					80.2	83.5	0.00
Zaire	DP	ZD025_D42	280.089		124.401			178.488			166.589			189.653					72.551		
Zaire	DP	ZD027_D0	223.9		139.9			171.4			154			175.1					83.2		0.00
Zaire	DP	ZD027_D42	237.89		124.396			175.405			163.393			162.449					82.35		
Zaire	DP	ZD039_D0	236.2		137.4			137.2			154			163					83.5		0.00
Zaire	DP	ZD039_D28	237.071		151.431			178.962			183.223			183.817					82.424		
Zaire	DP	ZD045_D0	244.6		142.9			175.6			154.1			162.1					83.5		0.00
Zaire	DP	ZD045_D42	253.449		124.242			174.493			151.408			167.6					82.36		
Zaire	DP	ZD048_D0	228		122.2			177.5			154			171.4					83.5		0.00
Zaire	DP	ZD048_D28	245.151		124.427			138.085			151.838			174.623					72.625		
Zaire	DP	ZD060_D0	234.3		123.6			178.7			160.7	185.8		162.8	169.1				83.5		0.00
Zaire	DP	ZD060_D35	263.35		124.125			169.358			166.882			186.599					80.148		
Zaire	DP	ZD067_D0	244.8		123			138.4			151.7			174.4					73.8		0.00
Zaire	DP	ZD067_D35	228.814	241.109	123.396	139.99		163.223			145.592	180.187		166.112					80.042	83.274	
Zaire	DP	ZD068_D0	244.7		141			138.1	180.6		150.9			175.2					73.8		0.00
Zaire	DP	ZD068_D35	214.24	247.595	120.265			175.456	184.655		167.577			162.374	168.787				80.027	83.259	
Zaire	DP	ZD080_D0	252.7		165.9	179		169.5			183.3			158.6					83.5		0.00
Zaire	DP	ZD080_D42	247.284		149.288			174.471			157.206			0					89.732		
Zaire	DP	ZD082_D0	228	241.6	124.3			159.3			160.3			174.5					80.3		0.98
Zaire	DP	ZD082_D28	228.447	234.632	124.414	139.028		160.101	163.079		119.144	160.358		167.389					80.073		
Zaire	DP	ZD088_D0	228		149.2			172.4			138.7			181.4					80.3		0.00
Zaire	DP	ZD088_D35	257.368		149.253			160.135			150.61			162.652					83.377		
Zaire	DP	ZD092_D0	228.2	240.3	138.8			168.3			145.6	151.7		162.4	171.6				83.5		0.07
Zaire	DP	ZD092_D28	239	249.5	140.6			171.1	180.3		132	150		0					78.753	82.034	
Zaire	AL	ZL302_D0	244.6		109.2			163			166.6			189.5					74	83.6	0.14
Zaire	AL	ZL302_D28	0		0			0			0			164.633					0		
Zaire	AL	ZL310_D0	213.918		0			140.751			164.304	167.52		0					0		0.00
Zaire	AL	ZL310_D14	218.403	226.808	124.408	137.954		171.305			158.259	160.284		168.788	193.118				89.741		
Zaire	AL	ZL315_D0	229.8		145.1			162.2			144.4			174.6					84.1		0.99
Zaire	AL	ZL315_D21	230.487		145.141			163.223			142.238	144.462		175.14					83.353		
Zaire	AL	ZL323_D0	221.9		145			140.2			150.9			175.2					80.3		0.00
Zaire	AL	ZL323_D21	241.231		149.291			160.132			180.144			190.199					82.362		
Zaire	AL	ZL332_D0	240.3		143.1			168.3			164.4			169.1					80.3	83.5	0.10
Zaire	AL	ZL332_D21	239.116	255.472	134.795			163.188	175.396		151.822			165.689	174.773				79.163	83.331	
Zaire	AL	ZL337_D0	252.7		153.4			171.4			151.9			181.5					90		0.00
Zaire	AL	ZL337_D28	222.627	239.083	137.812	164.659		163.215	169.04	178.665	154.158	166.855		159.608	171.867				80.112	83.349	
Zaire	AL	ZL339_D0	228.1		123.6			165.2			155.1			174.5					83.6		0.00
Zaire	AL	ZL339_D28	241.1		151.419			0			0			0					0		
Zaire	AL	ZL341_D0	234.4	244.6	138.2	151.7		159.1	168.4	171.4	154.2			171.5	190.2				80.3	83.5	0.85
Zaire	AL	ZL341_D21	235.056		151.493			160.169	169.378	172.489	155.181	164		156.506	168.771	171.642	190.02	193.041	80.051	83.284	
Zaire	AL	ZL343_D0	232.3		123.3			162.3			166.7			166.1					80.3		0.00
Zaire	AL	ZL343_D21	218.414		123.359			203.389			113.696			162.042					83.263		
Zaire	AL	ZL363_D0	224.1	244.6	143.1			168.4	171.4		144.5	158.3		162.8	174.8	180			80.3		0.98
Zaire	AL	ZL363_D21	224.754		143.084			172.423			145.418			162.657	175.164				80.09		
Zaire	AL	ZL365_D0	221.8	242.2	139.9			165.3	168.4		150.9	158.1		162.7	172.1				83.5	89.9	0.99
Zaire	AL	ZL365_D14	222.41		139.94			160.141	166.273		150.9	158.149		162.2	171.6				82.393	88.845	
Zaire	AL	ZL369_D0	221.8		140			168.4			144.7			158.9					80.3		0.00
Zaire	AL	ZL369_D21	238.6		123.422	145.044		181.602			161.312	167.623		180.108					83.03		
Zaire	AL	ZL370_D0	232.3		123.4			166.2			150.7			169.1					83.5		0.97
Zaire	AL	ZL370_D14	233.018		124.428			166.285	178.508		141.515	151.842		168.5	180.7				83.341		

Zaire	AL	ZL378_D0	225.1		139.5		169.4	172.4		139.4		168.4		80.4	83.6	0.99
Zaire	AL	ZL378_D21	227.91		138.813		172.452			139.269		169		80.18		
Zaire	AL	ZL379_D0	252.7	220	124.4	142	175.7			145.6		158.6		83.5		0.94
Zaire	AL	ZL379_D21	253.265		143.097		175.456			142.207	144.452	159.209		83.145		
Zaire	AL	ZL383_D0	225.9		124.2		166.1			161.2		167.8		83.5		0.00
Zaire	AL	ZL383_D21	210.104		138.917		160.218			163.451		164.291		83.237		
Zaire	AL	ZL384_D0	232.2	248.7	139.9		162.1	165.3		158.1	173.8	168.6	186.9	83.5		0.99
Zaire	AL	ZL384_D21	232.403		152.959		166.235			172.837		186.516		82.358		
Zaire	AL	ZL386_D0	226.1		136.8	145	174.5	178.5	189.7	144.6	157.2	138.2	171.7	77.1	83.5	0.00
Zaire	AL	ZL386_D28	261.349		149.277		175.202			140.951		161.456		80.064		
Zaire	AL	ZL391_D0	232.2		146.1		164.5			145.4		159.8	172	83.7		0.01
Zaire	AL	ZL391_D21	254.891		124.026	144.555	171.4			163.289		187.172		82.36		
Zaire	AL	ZL398_D0	240.3		164.7		177.5			167.7		172.1		83.5		0.00
Zaire	AL	ZL398_D28	226.684		132.722		184.609			151.786		178.272		83.3		
**Treatment Successes**																
Benguela	AL	BL182_D0	219.652		145.2		163.411			173.992		180.576		80.404		
Benguela	AL	BL189_D0	246.568		124.324		177.415			144.581		162.782		83.424		
Benguela	AL	BL205_D0	255.068		141.896		201.881			158.195		165.965		73.765		
Benguela	AL	BL211_D0	224.042		123		175.21			150.787		172.175		73.614		
Benguela	AL	BL219_D0	247		141		168.3	203		157.7		163.1	166	73.9	80.4	
Benguela	AL	BL228_D0	228.191		171.052		159.325			154.857		162.094		83.521		
Benguela	AL	BL247_D0	217.45		147.044		168.289			158.152		171.471		83.489		
Benguela	ASAQ	BQ601_D0	263.328		123.311		180.7			154.045		171.359		82.588		
Benguela	ASAQ	BQ625_D0	263.104		145.065		180.574			138.172		144.7		80.385		
Benguela	ASAQ	BQ652_D0	223.975	246.463	136.702		171.326			160.591	167.705	168.9		82.567		
Benguela	ASAQ	BQ684_D0	211.735	232.258	124.327	141.908	168.381			158.175		162.201	195.955	73.828		
Lunda Sul	DP	LD453_D0	223.929		142.952		168.266			157.956		165.872		83.503		
Lunda Sul	DP	LD466_D0	219.81	249.9	138.814		165.255	171.383		148.684	164.509	162.825	168.387	80.264		
Lunda Sul	DP	LD467_D0	246.829		145.248		172.379			153.983		171.708	174.569	83.604		
Lunda Sul	DP	LD469_D0	230.27		136.806		174.415			183.37		162.211		80.299		
Lunda Sul	DP	LD471_D0	253.113		141.032		169.091			177.122		162.952		83.588		
Lunda Sul	DP	LD476_D0	236.436		124.314		165.279			170.678		193.016		80.264		
Lunda Sul	DP	LD482_D0	221.688		142.945		165.274	177.519		158.322		180.458		80.276		
Lunda Sul	DP	LD486_D0	224.071	246.764	124.345	142.99	171.432			164.453		159.326	189.81	73.809		
Lunda Sul	DP	LD494_D0	223.95		124.357	143.008	165.237			164.418	183.311	172.001		83.403		
Lunda Sul	DP	LD502_D0	219.947	248.809	147.037		165.273	183.624		150.926	164.538	171.463	186.765	80.291		
Lunda Sul	DP	LD513_D0	248.695		139.797		160.139			158.136		162.143		80.197		
Lunda Sul	DP	LD523_D0	252.967		125.292		169.334			170.622		162.138		83.549		
Lunda Sul	DP	LD526_D0	219.84	236.378	157.423		165.247	174.428		173.814		174.573		83.457		
Lunda Sul	DP	LD534_D0	223.884		132.595		165.243	189.888		138.333	147.825	189.228	196.443	83.439		
Lunda Sul	DP	LD540_D0	231.4		145.2		166.5			157.1		166.1		83.6		
Lunda Sul	DP	LD553_D0	211.474		143.008		177.077			154.969		169.034				
Zaire	DP	ZD034_D0	213.656	230.111	147.118		159.123	166.205		151.848	176.936	168.938	189.594	83.501		
Zaire	DP	ZD051_D0	222.938	231.257	124.32		159.08			154.991		168.814		80.283		
Zaire	DP	ZD090_D0	232.244		138.85		174.356			157.219		186.332		83.492		
Zaire	AL	ZL333_D0	240.617		138.618		165.543			179.24		168.247		70.42		
Zaire	AL	ZL377_D0	228.141		137.875		177.524	183.645		142.53	154.055	156.373		80.302		
Zaire	AL	ZL328_D0	229.1		123.5		184.7			161.1		161.4		83.5		
Zaire	AL	ZL355_D0	267.3		123.3		163			170.7		181.4		83.5		
Zaire	AL	ZL404_D0	219.9		145.1		171.4			144.6		174.6		83.6		
